# Government Regulation, Executive Overconfidence, and Carbon Information Disclosure: Evidence From China

**DOI:** 10.3389/fpsyg.2021.787201

**Published:** 2021-12-10

**Authors:** Ren He, Yanduo Cheng, Mingdian Zhou, Jing Liu, Qing Yang

**Affiliations:** ^1^School of Maritime Economics and Management, Dalian Maritime University, Dalian, China; ^2^School of Economics, Fudan University, Shanghai, China; ^3^School of Management Engineering, Anhui Institute of Information Technology, Wuhu, China

**Keywords:** carbon information disclosure, government regulation, executive overconfidence, low-carbon pilot provinces and cities, climate change

## Abstract

Climate change has put countries around the world under great pressure to reduce greenhouse gas emissions. Chinese government has proposed that China will strive to peak carbon dioxide emissions before 2030 and achieve carbon neutrality before 2060. A low-carbon lifestyle is becoming a new trend in China. Therefore, the products of firms that actively respond to climate change are more popular for consumers in China. In the Internet era, the carbon information disclosed by firms has become an important way for consumers to understand the behavior of firms in responding to climate change. In the existing literature on the influencing factors of carbon information disclosure, the psychological factors of executives are seldom investigated. Using a sample of Chinese listed firms in low-carbon pilot provinces and cities during the period of 2015–2019, this study explores the influence of government regulation and executive overconfidence on the quality of carbon information disclosure. The results show that government regulation has a significantly positive impact on the quality of carbon information disclosure. The results also reveal that executive overconfidence negatively affects the quality of carbon information disclosure. Moreover, executive overconfidence negatively moderates the relationship between government regulation and the quality of carbon information disclosure. Our findings make a significant contribution to the role of executive’s psychological factors in firm’s behaviors and provide new insights and policy implications for government, firms, consumers, and other stakeholders.

## Introduction

During the industrial revolution, capitalism was liberated from the handicraft industry and entered the stage of transition to the machine industry. Since then, major changes have taken place in the economic development mode of various countries, and the increase in production efficiency has promoted the rapid development of industry. However, industrial development has brought many adverse effects to the environment at the same time. For example, the large-scale use of fuels has caused carbon emissions level to reach new highs, which in turn led to global climate change, and extreme weather appeared more and more frequently (Todea et al., 2013). The global climate is experiencing significant warming, and the harm of the greenhouse effect is appearing gradually. How to alleviate climate change scientifically and effectively has aroused widespread concern in the political and scientific circles of the world. In order to combat the detrimental consequences of the climate change phenomenon, in 2016, by signing the Paris Agreement, governments from around the world established specific environmental targets to reduce the level of greenhouse gas emissions and to limit global warming to well below 2, preferably to 1.5°C, compared to pre-industrial levels (Siri and Zhu, 2019).

As the largest developing country in the world, China is actively making unremitting efforts to address the issue of climate change. China made a significant US. China Joint Presidential Statement on Climate Change on 25 September 2015, pledging to lower carbon dioxide emissions per unit of GDP by 60–65% from 2015 level by 2030 (Liu and Li, 2019). Chinese economic development goals have changed from purely pursuing GDP growth to pursuing the balanced development of all aspects of the national economy. In order to fulfill its promises as scheduled, Chinese government attaches great importance to the control of domestic carbon emissions and vigorously advocates green and low-carbon production methods and lifestyle, and China has issued a series of low-carbon policies, such as the “Comprehensive Working Programme on Energy Conservation and Emission Reduction for the 13th Five-Year Plan Period” and the “Interim Rules for Carbon Emissions Trading Management.”

Firms are an important source of carbon emissions, and their carbon emissions are more likely to be concerned by the government and the public. The governments around the world implement various policies to control or reduce carbon emissions of firms, such as carbon tax and green subsidies (Martelli et al., 2020). Firms’ carbon emissions data and low-carbon behaviors are shown through the disclosure of carbon information. Firms can regularly release carbon information through the media to create a good image of green and low-carbon to the local governments and attract consumers to buy their products. With the development of the Internet and big data technologies, the accounting, management, and disclosure of firms’ carbon emissions data are becoming more efficient and low-cost. Therefore, more and more firms are willing to use carbon information disclosure as a marketing strategy. In this context, it is of great significance to study the impact of government regulation on the disclosure of firms’ carbon information.

According to the theory of legitimacy, government regulatory can limit firms’ behavior and prevent firms from sacrificing environmental performance in order to maximize profits (Hafezi and Zolfagharinia, 2018). In addition to the external factors, such as government regulation, internal factors also affect the behaviors of firms. Existing studies rarely explore the impact of government regulation on firms’ carbon information disclosure behavior. Although existing studies have investigated the effects of some internal factors, such as firms’ characteristics and corporate governance factors, on firms’ carbon information disclosure (Ben-Amar et al., 2017; Kouloukoui et al., 2019; He et al., 2021a,b), few studies examine the effects of executives’ characteristics on firms’ carbon information disclosure.

Based on legitimacy theory and behavioral finance theory, this study analyzes the impacts of government regulation and executive overconfidence on the quality of firms’ carbon information disclosure and explores the moderating role of executive overconfidence in the relationship between government regulation and the quality of firms’ carbon information disclosure. This study makes the following contributions to the existing literature. First, to the best of our knowledge, this study is the first to empirically investigate whether executive overconfidence may influence the quality of firms’ carbon information disclosure. Existing research has explored the influence of executive overconfidence on corporate performance, decision making, and corporate governance, but they provide little evidence on the impact of executive overconfidence on firms’ carbon information disclosure.

Second, this study extends existing research on government regulation. The impact of government regulation has always been the hot topic of academic research. Existing research on government environmental regulation mainly focuses on the type, impacts, and effectiveness of government regulation. However, there is no consistent research conclusion on its impact on firms’ carbon information disclosure.

Third, to the best of our knowledge, this study is the first to explore the moderating role of executive overconfidence in the governance effect of government regulation. Overconfident executives probably make irrational decisions, which in turn will affect the firms’ strategic choices, but the previous literature provides little evidence on the influence of executive overconfidence on the governance effect of government regulation. This study finds that executive overconfidence can negatively moderate the relationship between government regulation and the quality of carbon information disclosure.

Finally, focusing on the Chinese context, China has not a mandatory carbon information disclosure policy, and the carbon information disclosed by firms is voluntary. Thus, executives have a lot of choice when they make carbon information disclosure decisions. As the Chinese government is increasingly paying attention to environmental issues, such as climate change, this study can better investigate the influence of government regulation and executive overconfidence on carbon information disclosure.

The other parts of this paper are arranged as follows: Section “Literature Review” provides the literature review. Section “Theory and Hypotheses” proposes the theory and hypotheses. Section “Materials and Methods” discusses the empirical research design. Section “Results” presents the analysis of the empirical results, and Section “Conclusion, Implications, and Limitations” presents the conclusion, implications, and limitations.

## Literature Review

### Government Regulation and Environmental Information Disclosure

In the process of economic development, environmental pollution has caused negative spillover effects, which have a significant impact on sustainable development. In order to achieve the dual goal of coordinated development of economy and environment, the government will adjust corresponding economic activities and implement environmental regulation. The existing research on government environmental regulation mainly focuses on the type, impacts, and effectiveness of environmental regulation (Ziegler et al., 2012; Cheng et al., 2017; Liu and Li, 2019). For example, existing studies have explored the effects of government regulation on firms’ environmental performance (Akram et al., 2018; Lu, 2020; Wang et al., 2020), green technology innovation (Kammerer, 2009; Ai et al., 2021), sustainability, and corporate social responsibility strategies (Schmutz et al., 2020).

In most countries, especially in developing countries, firms’ environmental information disclosure is voluntary. If there is no related policy issued by the government, the environmental information disclosed by firms will lack comparability and practicality. Strengthening environmental regulation can significantly improve the quality of firms’ environmental information disclosure (Buhr and Freedman, 2001). The main reason for firms to disclose environmental information is the pressure from the government, shareholders, and other stakeholders. Government regulation, through the formulation of systems and rules, evaluates the corporate environmental responsibility status, and forms a restraining mechanism for decision making (Othman and Ameer, 2010). Government policies are an important motivation for firms’ willingness to disclose carbon information (Tauringana and Chithambo, 2015).

However, the results of existing studies are inconsistent. Stanny (2013) found that when the regression model includes a previous disclosure variable, the regulatory factor becomes irrelevant. Confronting government environmental regulation, firms have either no behavioral response, or just a symbolic disclosure improvement, and the information disclosed is indiscriminate and invalid. The information disclosed may lack credibility, reliability, and be limited or misleading to information users (Tauringana and Chithambo, 2015). In addition, existing research often explores the combined effects of government regulation and other factors on environmental information disclosure, such as executives’ political connections (Luo et al., 2017), which may also lead to inconsistent conclusions on the impact of government regulation on firms’ environmental information disclosure.

### Executive Characteristics and Environmental Information Disclosure

Existing studies on the impact of executive characteristics mostly focus on firm behavior, financial performance, and innovation, there are only a few studies focus on firms’ environmental performance and information disclosure. The executive characteristics related to firm’s environmental performance and information disclosure that have been explored are mostly physiological and social characteristics, such as age (Li et al., 2019), tenure of office (Lewis et al., 2014; Suárez-Rico et al., 2018), and educational background (Slater and Dixon-Fowler, 2010; Lewis et al., 2014). Overconfidence is an important psychological characteristic of executives. However, the impact of executive overconfidence on firms’ environmental information disclosure is paid little attention.

Previous research mainly focuses on the economic consequences of executive overconfidence. Compared with rational executives, overconfident executives frequently implement mergers and acquisitions, especially when they have sufficient internal funds. As the level of overconfidence of executives increases, mergers and acquisitions also increase, especially diversified mergers. But the benefits of mergers and acquisitions implemented by overconfident executives will be lower (Malmendier and Tate, 2005; Andreou et al., 2019). Overconfidence can lead to behavioral deviations of executives. Overconfident or optimistic managers will overestimate future investment returns, overestimate the net present value (NPV) of investment projects, and invest in projects with NPV less than 0, leading to distortions in investment, resulting in over-investment (Heaton, 2002). Executives’ optimism about their own capabilities and the future of firms makes them believe that external financing costs are too high, and they tend to use internal funds and debt capital to reduce investment costs. Therefore, they are likely to cause under-investment (Landier and Thesmar, 2009; Huang et al., 2011). As overconfident executives advocate freedom and liberation, are more radical, and have the courage and determination to carry out high-input, high-risk, and high-uncertain R&D activities, they are willing to accept challenges and increase R&D investment, enhance the firms’ learning and absorptive capacity, enable the firm to maintain a high level of competition, and thus improve the firms’ performance significantly (Hirshleifer et al., 2012; Hai et al., 2020).

The overconfidence of executives will also affect firms’ information disclosure behavior. Overconfident managers prefer to publish earnings forecasts. The results of the forecasts are optimistic, and the scope of the forecast is narrower (Hribar and Yang, 2016). Because of the tendency to ignore the importance of internal control over financial reporting, overconfident managers will have a negative impact on the firms’ infrastructure investment in implementing effective financial reporting information systems. The higher the degree of manager’s overconfidence, the greater the possibility of restatement of the financial report (Lee, 2015). But there is little evidence on the impact of executive overconfidence on firms’ environmental information disclosure, especially carbon information disclosure.

Based on the above, this study explores the impact of government regulation, executive overconfidence on the quality of firms’ carbon information disclosure. This study has theoretical and practical values for studying the influencing factors of firms’ carbon formation disclosure and for improving the governance effect of government regulation on firms’ environmental information disclosure.

## Theory and Hypotheses

### Government Regulation and the Quality of Carbon Information Disclosure

The concept of “legitimacy” was introduced into the field of political science and studied systematically by Max Weber. Since then, “legitimacy” has gradually become the core concept and mainstream paradigm of modern political science and sociology. In a social activity system composed of command and obedience, the normal operation of the social activity system requires the establishment and cultivation of a universal belief in the meaning of its existence, that is, the legitimacy of existence. Suchman (1995) used the concept of legitimacy to study organizational legitimacy. Suchman (1995) gave a more authoritative definition of legitimacy. Legitimacy is a generalized perception or assumption that the actions of an entity are desirable, proper, or appropriate within some socially constructed system of norms, values, beliefs, and definitions. Legitimacy is a key resource for the survival of a firm, and it represents the legitimacy of the degree of cultural support obtained from the environment. Therefore, the firm needs legitimacy and needs to be recognized by the society for its goals, in order to develop its activities and better obtain other types of resources. If an organization does not adhere to the goals, methods and results recognized by the society, the operation of the organization will not succeed, or even the organization cannot survive. In addition, legitimacy is a dynamic resource that can change over time, so firms need to develop different strategies to obtain, repair, or maintain their legitimacy.

Legitimacy exists in people’s consciousness, and human perception has a major impact on the perception of legitimacy. Therefore, information disclosure has become a basic element of the legitimacy strategy (Breton and Côté, 2006; Magness, 2006). When legitimacy is threatened, firms often increase the quality of information disclosure to gain legitimacy status. Even if a firm adapts to society’s expectations of the environment, if the firm fails to show its compliance by disclosing environmental information, its legitimacy will also be threatened. Therefore, the information disclosure of firms can effectively maintain their own legitimacy. By changing their behaviors, they can show that their own values are consistent with social values in order to be recognized by the public (Deegan and Rankin, 1996). Factors, such as local legal system environment, economic development level, environmental protection awareness, and other factors, affect the local government’s regulation of firms’ environmental information disclosure. However, there are differences in regulatory powers in different regions (Liu and Li, 2019). The more local governments pay attention to the disclosure of environmental information, the greater the regulation of firms’ environmental information disclosure will be. Under different levels of environmental regulation, firms will have different enthusiasm for carbon information disclosure, and different behavioral tendencies will appear in firms’ decision making, resulting in different quality of carbon information disclosure. Therefore, we formulate the following hypothesis:

*Hypothesis 1*: Government regulation positively affects the quality of carbon information disclosure.

### Executive Overconfidence and the Quality of Carbon Information Disclosure

For the first time, Burrell (1951) combined psychology and finance to do financial decision making research, and is regarded as the pioneer of behavioral finance. The most important psychological deviation in behavioral finance is overconfidence (Nkukpornu et al., 2020). Executives have the right to speak in important decisions of the firm and can decide major activities, such as investment, financing, mergers and acquisitions. Carbon information disclosure is also one of the executive’s decisions. When making carbon information disclosure decisions, executives will balance benefits against risks, as well as benefits against costs. Carbon information disclosure cannot obtain economic benefits in the short term, and overconfident managers have higher risk appetite, which will reduce the firms’ willingness to disclose carbon information to a certain extent (Tang et al., 2015). Overconfident executives are likely to underestimate the necessity or ability of stakeholders to provide resources, and thus neglect to exchange benefits with their stakeholders through carbon information disclosure to facilitate the further development of the firm. Overconfident executives may also overestimate the total amount of potential resources owned by the firm, overestimate their own ability to deal with the problem of insufficient firm resources (Aabo et al., 2021). Overconfident executives believe that they can control the development of things, so when making decisions, they tend to adopt more risky and aggressive methods, and the level of risk taking of their firms will be higher (Schumacher et al., 2020). Compared with overconfident executives, non-overconfident executives often disclose more carbon information because of risk aversion. Therefore, we formulate the following hypothesis:

*Hypothesis 2*: Executive overconfidence negatively affects the quality of carbon information disclosure.

### Government Regulation, Executive Overconfidence, and the Quality of Carbon Information Disclosure

The negative effects of the environmental issues on the economy have become increasingly serious in China. Macroeconomic policies have focused on the development of low-carbon economy and paid attention to environmental issues. The concept of “low carbon” has gradually become popular. The government has begun to discover the importance of environmental information disclosure and has issued a series of laws, regulations to promote firms’ environmental information disclosure. Mandatory policies require firms to meet the required environmental standards and penalize firms that fail to meet the standards. These penalties will increase the pollution cost of the firm and even affect the survival and development of the firm. Furthermore, the incentive system is introduced into the firms, and the firm that actively meets the standards and improves the environmental information disclosure is given rewards and government subsidies or preferential policies (Huang and Chen, 2015). Under such policies, government, investors, and the public are paying more and more attention to carbon emissions, the legality of carbon emissions has become an important condition for the sustainable development of firms. But overconfident executives rely on their own abilities too much, and ignore the constraints of objective conditions, they will make irrational decisions. The upper echelon theory believes that confronting complex internal and external environments, managers cannot perceive environmental information at all levels and cannot have a comprehensive and objective understanding. Therefore, the personal characteristics of managers will affect their personal style and accounting behavior to a certain extent. They use their cognitive foundation and values to filter information, interpret the situation and make decisions, and then affect the strategic decisions and behaviors of firms (Hambrick and Mason, 1984). Overconfident managers usually believe that they have enough information to deal with future risk factors, believe that their judgments are correct, and thus overestimate the probability of success and underestimate the probability of failure (March and Shapira, 1987). Overestimating their own capabilities, and underestimating the risks of objective constraints, overconfident managers tend to ignore relevant policies that affect firms’ information disclosure strategy.

Although the establishment of a carbon information disclosure system is important for the establishment and development of the carbon emission trading system in China, the quality of firms’ carbon information disclosure is not high, and it is still in the stage of non-standard voluntary disclosure. Regarding the carbon information disclosure, executives have many choices. They can freely adjust the qualitative or quantitative carbon information disclosures according to the firm’s conditions. As a result, the executive overconfidence is likely to inhibit the role of government regulation in improving the quality of firms’ carbon information disclosure. Therefore, we formulate the following hypothesis:

*Hypothesis 3*: Executive overconfidence negatively moderates the relationship between government regulation and the quality of carbon information disclosure.

## Materials and Methods

### Sample and Data Sources

The initial samples of our study include all A-share listed firms in 10 low-carbon pilot provinces and cities in China between the years 2015 and 2019, which include Guangdong, Liaoning, Hubei, Shaanxi, Yunnan, Tianjin, Chongqing, Hainan, Beijing, and Shanghai. The above 10 provinces and cities basically cover the regions of South China, Northeast China, Central China, Northwest China, North China, Southwest China, and East China, which can comprehensively reflect the regional distribution of the quality of firms’ carbon information disclosure in China. Firms in financial industry, and firms with missing data and marked with ST or ^*^ST are excluded from samples. The final samples include 4,620 firm-year observations. The carbon information is derived from the firms’ annual reports, social responsibility reports, and environmental reports or sustainability reports. The other data are from the CSMAR database.

### Variable Definition

#### Dependent Variable

The dependent variable is the quality of carbon information disclosure. At present, there is no authoritative carbon information disclosure framework in China. We adopt the carbon information disclosure index (CDI) used in previous research to measure the quality of firm’s carbon information disclosure (Liu et al., 2017). We use content analysis to evaluate the total score of 22 items. A score of one is assigned if a disclosure is related to items in CDI. The specific scoring criteria are shown in Table 1.

**Table 1:** Items included in the carbon information disclosure index.

ID		Category/item
CC		Climate change–related risks, opportunities and actions
1	CC1	Risks associated with climate change
2	CC2	Description of the actions initiated or planned as a result of identification of risks associated with climate change
3	CC3	Opportunities associated with climate change
4	CC4	Actions initiated or planned as a result of identification of opportunities associated with climate change
GHG		GHG emission accounting
5	GHG1	Methodology used to calculate GHG emissions
6	GHG2	External verification/assurance status that applies to GHG emissions
7	GHG3	Total GHG emissions
8	GHG4	Breakdown of GHG emissions
9	GHG5	GHG emission intensity
10	GHG6	Strategies to reduce GHG emissions
11	GHG7	GHG emission reduction plans
12	GHG8	GHG emission intensity reduction
EC		Energy consumption accounting
13	EC1	Total energy consumption
14	EC2	Breakdown of energy consumption
15	EC3	Total renewable energy consumption
16	EC4	Breakdown of renewable energy consumption
17	EC5	Strategies to increase renewable energy use
18	EC6	Strategies to reduce energy use
ACC		Climate change–related governance and accountability
19	ACC1	Board committee responsible for climate change risk management
20	ACC2	How the board reviews progress on firms carbon performance
21	ACC3	Incentives for managing GHG emissions and energy use
22	ACC4	Staff development programs to encourage reduction of emissions and energy use

#### Independent Variables

One independent variable is government regulation. In order to reflect the environmental regulation status in different regions in China, we adopt the Pollution Information Transparency Index (PITI) to measure government regulation. PITI is published by the Institute of Public and Environmental Affairs (IPE) and the Natural Resources Defense Council (NRDC), and is under the guidance of “Regulations on Disclosure of Government Information” and “Measures for the Disclosure of Environmental Information” published by Chinese government. PITI is used to assess the level of pollution source information disclosure of environmental protection divisions of local governments in 113 Chinese cities (Tian et al., 2016). The number of cities assessed has increased to 120 from 2013. As published by a third-party non-governmental organization, PITI can objectively reflect the implementation of relevant regulations of environmental information disclosure by local governments.

The other independent variable is executive overconfidence. The measures of this irrational psychological characteristic of executives used in previous research include executive earnings forecast bias (Huang and Yang, 2019), stock option (Banerjee et al., 2018; Lee et al., 2018), and mainstream media evaluation (Brown and Sarma, 2007; Hribar and Yang, 2016). To a certain extent, the level of salary reflects the self-confidence of senior managers. The senior managers are more likely to be overconfident when their abilities and salaries are higher. Moreover, the salary is relatively less interfered by system and industry factors, and can better reflect senior managers’ ability and behavioral characteristics. Therefore, we use the salaries to measure executive overconfidence. Hayward and Hambrick (1997) use relative salary to measure CEO overconfidence. In this study, we also adopt relative salary to measure the degree of executive overconfidence. The relative salary is the sum of the salaries of the top three highest paid executives divided by the total salaries of all executives.

#### Control Variables

According to previous research, the factors we control include institutional investors (INS), creditor pressure (LOANS), political connection (PC), profitability (ROA), organizational structure (ORG), leverage (DEB), analyst coverage (ANALYST), duality (DUAL), educational background (MAJOR), board independence (INDE), and industry (IND) (Cho et al., 2017; Liu et al., 2020; Xiang and Birt, 2020). The industry is a dummy variable that equals to 1 if the firm belongs to a high carbon industry and 0 otherwise. The high carbon industries in this study include the following 10 industries: Production and supply of electric power and heat, Oil and gas extraction, Coal mining and washing, Chemical raw materials and chemical products manufacturing, Petroleum processing, coking and nuclear fuel processing, Ferrous metal smelting and rolling processing, Non-metallic mineral products, Non-ferrous metal smelting and rolling processing, Papermaking and paper products, Ferrous metal mining and dressing. The definitions of variables are shown in Table 2.

**Table 2:** Variable definitions.

Variables	Symbols	Descriptions
Quality of carbon information disclosure	CDI	Carbon Information Disclosure Index
Government regulation	PITI	Pollution Information Transparency Index
Executive overconfidence	OC	The sum of the salaries of the top three highest paid executives/total salaries of all executives
Institutional investors	INS	Institutional investors’ shareholding ratio
Creditor pressure	LOANS	The ratio of bank loans to total liabilities
Political connection	PC	Percentage of executives with political background
Profitability	ROA	The ratio of net profit to total assets
Organizational structure	ORG	A dummy variable that equals to 1 if the firm has an environment committee or a dedicated environment division and 0 otherwise
Leverage	DEB	The ratio of total debt to total assets
Analyst coverage	ANALYST	The number of analysts who made a profit forecast for a firm during the year
Duality	DUAL	A dummy variable that equals to 1 if the CEO is the board’s chairman and 0 otherwise
Educational background	MAJOR	A dummy variable that equals to 1 if the major of the chairman or CEO is business or economic management and 0 otherwise
Board independence	INDE	Proportion of independent directors in the board of directors
Industry	IND	A dummy variable that equals to 1 if the firm belongs to a high carbon industry and 0 otherwise
Year	YEAR	Dummies for each of the 5 years from 2015 to 2019 inclusive

#### Model Design

In this study, ordinary least squares regression analysis is used to test our hypotheses. Model 1 is used to test the impact of government regulation on the quality of carbon information disclosure. Model 2 is used to test the impact of executive overconfidence on the quality of carbon information disclosure. Model 3 is used to test the impact of executive overconfidence on the relationship between government regulation and the quality of carbon information disclosure.


(1) $$\begin{array}{l} CDI=\alpha +\beta_{1}PITI+\beta_{2}INS+\beta_{3}LOANS+\beta_{4}PC+\beta_{5}ROA\\ +\beta_{6}ORG+\beta_{7}DEB+\beta_{8}ANALYST+\beta_{9}DUAL\\ +\beta_{10}MAJOR+\beta_{11}INDE+\beta_{12}IND+\sum \beta_{i}YEAR_{i}+\varepsilon \end{array}$$

(2) $$\begin{array}{l} CDI=\alpha +\beta_{1}OC+\beta_{2}INS+\beta_{3}LOANS+\beta_{4}PC\\ +\beta_{5}ROA+\beta_{6}ORG+\beta_{7}DEB+\beta_{8}ANALYST\\ +\beta_{9}DUAL+\beta_{10}MAJOR+\beta_{11}INDE\\ +\beta_{12}IND+\sum \beta_{i}YEAR_{i}+\varepsilon \end{array}$$

(3) $$\begin{array}{l} CDI=\alpha +\beta_{1}PITI+\beta_{2}PITI*OC+\beta_{3}OC+\beta_{4}INS\\ +\beta_{5}LOANS+\beta_{6}PC+\beta_{7}ROA+\beta_{8}ORG+\beta_{9}DEB\\ +\beta_{10}ANALYST+\beta_{11}DUAL+\beta_{12}MAJOR\\ +\beta_{13}INDE+\beta_{14}IND+\sum \beta_{i}YEAR_{i}+\varepsilon \end{array}$$

## Results

### Descriptive Statistics

#### Descriptive Statistics of Carbon Information Disclosure Index

Table 3 provides the descriptive statistics of CDI. The results show that the quality of firms’ carbon information disclosure in China is relatively low. In most years of the sample period, the average scores of firms’ CDI are less than 1. Further analysis shows that 75.35% of the samples’ CDI scores are 0 or 1, 23.57% of the samples’ CDI scores are between 2 and 7, only 1.08% of the samples’ CDI scores are higher than 8, which indicates that most of the sample firms have not disclosed or disclosed a little carbon information. In recent years, the Chinese government has issued a series of energy-saving and emission-reduction policies, and firms have paid more attention to low-carbon development.

**Table 3:** Descriptive statistics of CDI.

Year	2015	2016	2017	2018	2019
Observation	881	976	1,029	844	890
Mean	0.9015	0.9529	0.9971	1.1576	0.9978
Standard deviation	1.5968	1.7055	1.7840	1.9756	1.6456
High carbon industries	1.3636	1.6311	1.7523	2.0294	1.5047
Non-high-carbon industries	0.8427	0.8729	0.9076	1.0377	0.9285
State-owned firms	1.5723	1.6436	1.7725	1.9233	1.6888
Non-state-owned firms	0.4816	0.5456	0.5869	0.7353	0.7411

The results of Table 3 show a steadily growing trend of firms’ CDI, indicating that the awareness of firms’ carbon information disclosure has been continuously increasing. However, firms’ CDI declined in 2019. This may be due to the outbreak of COVID-19 at the beginning of 2020, which has caused many interferences in firms’ information disclosure. Emergencies, most of which are challenges to the assumption of firm’ sustainable operation, will cause firm’s business crisis. Confronting crisis, abnormal changes of firms’ internal and external factors, operating rules, and development environment will threaten firms’ survival in severe cases. Compared with the situation before the emergency, firms’ information disclosure will change. The standard deviation of firms’ CDI also shows a growing trend, indicating that the gap in sample firms’ carbon information disclosure is more obvious.

The results of Table 3 show that there are obvious differences in the quality of carbon information disclosure of firms with different industries and ownership. The average CDI of firms in high carbon industries is higher than that of firms in non-high-carbon industries, which meets the theory of legitimacy. Firms in high carbon industries have high carbon emissions and need to disclose more carbon information to manage the legality of firms and to reduce or avoid penalties related to environmental pollution. The average CDI of state-owned firms is higher than that of non-state-owned firms, which indicates that state-owned firms are more affected by government intervention. State-owned firms will implement low-carbon policies and disclose carbon information more actively.

#### Descriptive Statistics of Other Variables

Table 4 shows the descriptive statistics of independent variables and control variables. The mean of government regulation, which is measured by PITI, is 64.19, with a minimum of 16.8 and a maximum 80.8, indicating that there is a large gap in government regulation between different regions in China. The mean of executive overconfidence (OC) is 0.444, indicating that the salaries of the top three highest paid executives accounts for approximately half of the total salaries of all executives in China. The mean of political connection (PC) is 0.042, indicating that there are only about 4% of the sample firms having political connection, and the degree of political connection of firms is not high in China. The mean of profitability (ROA) is 0.0437, with a minimum of −1.068 and a maximum of 0.384, indicating that most firms’ profitability is positive. The mean of organizational structure (ORG) is 0.0277, indicating that only 2.77% of the sample firms have established an environment committee or a dedicated environment division, and governance structure of firms in China needs to be improved. The mean of duality (DUAL) is 0.301, indicating that the CEO and the board’s chairman are the same person in about 30% of sample firms. The mean of educational background (MAJOR) is 0.535, indicating that more than half of the executives have got a degree in economics or management in China. The mean of industry (IND) is 0.113, indicating that there are about 11% of listed firms are in high carbon industries in low-carbon pilot provinces and cities in China. Table 4 also shows that the data of institutional investors (INS), creditor pressure (LOANS), leverage (DEB), analyst coverage (ANALYST), and board independence (INDE) are quite different, but overall, most of the data are relatively stable.

**Table 4:** Descriptive statistics of variables.

Variable	Obs	Mean	Std.Dev.	Min	Max
PITI	4,620	64.19	12.02	16.80	80.80
OC	4,620	0.444	0.131	0	1
INS	4,620	0.0679	0.0662	0	0.625
LOANS	4,620	0.246	0.206	0	0.917
PC	4,620	0.0420	0.0647	0	0.588
ROA	4,620	0.0437	0.0695	−1.068	0.384
ORG	4,620	0.0277	0.164	0	1
DEB	4,620	0.426	0.201	0.0174	1.345
ANALYST	4,620	9.960	10.04	1	75
DUAL	4,620	0.301	0.459	0	1
MAJOR	4,620	0.535	0.499	0	1
INDE	4,620	0.382	0.0611	0	0.800
IND	4,620	0.113	0.316	0	1

#### Correlation Analysis

Table 5 shows the Pearson correlations for all the dependent, independent, and control variables. It is evident that the quality of carbon information disclosure is positively correlated with government regulation and is negatively correlated with executive overconfidence, as expected. There are also strong correlations between several control variables. However, none of the correlations exceeded 0.5. Table 6 shows that the values of variance inflation factor (VIF) are less than 2, and the values of tolerance are greater than 0.7, which indicates that multicollinearity is less likely to be a problem for our analyses.

**Table 5:** Correlation analysis.

Variable	CDI	PITI	OC	INS	LOAN	PC	ROA	ORG	DEB	ANALYST	DUAL	MAJOR	INDE	IND
CDI	1.000													
PITI	0.077^***^	1.000												
OC	−0.166^***^	−0.001	1.000											
INS	0.070^***^	−0.037^**^	−0.055^***^	1.000										
LOANS	0.077^***^	−0.080^***^	−0.049^***^	0.011	1.000									
PC	0.070^***^	−0.022	0.037^**^	0.018	0.062^***^	1.000								
ROA	−0.013	−0.003	0.080^***^	0.068^***^	−0.206^***^	−0.017	1.000							
ORG	0.145^***^	0.020	−0.028^*^	0.024^*^	0.003	0.025^*^	0.032^**^	1.000						
DEB	0.239^***^	−0.037^**^	−0.163^***^	0.106^***^	0.405^***^	0.109^***^	−0.309^***^	0.015	1.000					
ANALYST	0.197^***^	0.084^***^	−0.063^***^	0.406^***^	−0.077^***^	0.037^**^	0.229^***^	0.064^***^	0.073^***^	1.000				
DUAL	−0.148^***^	0.041^***^	0.138^***^	−0.034^**^	−0.072^***^	−0.007	0.046^***^	−0.042^***^	−0.170^***^	−0.038^**^	1.000			
MAJOR	0.050^***^	0.009	0.009	0.040^***^	0.078^***^	0.095^***^	−0.015	0.017	0.106^***^	0.036^**^	−0.189^***^	1.000		
INDE	0.037^**^	0.025^*^	0.089^***^	0.001	−0.009	0.041^***^	−0.004	−0.047^***^	0.012	0.055^***^	0.105^***^	−0.020	1.000	
IND	0.134^***^	−0.076^***^	−0.042^***^	−0.023	0.199^***^	0.004	−0.013	0.007	0.063^***^	−0.002	−0.094^***^	−0.007	−0.005	1.000

^∗∗∗^*p < 0.01, ^∗∗^p < 0.05, and ^∗^p < 0.1*.

**Table 6:** Variance inflation factor test for variables.

Variable	VIF	Tolerance
PITI	1.20	0.8322
OC	1.06	0.9409
INS	1.25	0.8004
LOANS	1.27	0.7847
PC	1.03	0.9731
ROA	1.20	0.8302
ORG	1.01	0.9874
DEB	1.39	0.7192
ANALYST	1.35	0.7386
DUAL	1.10	0.9096
MAJOR	1.06	0.9405
INDE	1.03	0.9730
IND	1.06	0.9446

#### Regression Results

The regression results are shown in Table 7. The coefficient of PITI in Model 1 is positive and significant at the 1% level, which indicates that the positive relationship between government regulation and the quality of carbon information disclosure is significant, and the government regulation can promote the quality of carbon information disclosure. This result confirms Hypothesis 1. This is consistent with the previous analysis. The survival and development need firms to comply with the regulations and policies developed by the government. Government’s emphasis on environmental protection and governance promotes firms’ carbon information disclosure by making firms to show their legitimacy. Therefore, an appropriate increase in government environmental regulation will have a significant impact on improving the quality of carbon information disclosure. The coefficient of OC in model 2 is negative and significant at the 1% level, indicating that the higher the degree of executive overconfidence, the lower the quality of carbon information disclosure. This result confirms Hypothesis 2. The result of Model 3 shows that the coefficient of the interaction term between PITI and OC is significantly negative, which indicates that executive overconfidence has a negative moderating effect on the relationship between government regulation and the quality of carbon information disclosure. This result confirms Hypothesis 3.

**Table 7:** Multiple regression results.

Variable	CDI		
	Model 1	Model 2	Model 3
PITI	0.013^***^ (6.16)		0.013^***^ (5.79)
OC		−1.533^***^ (−8.16)	−1.511^***^ (−8.08)
PITI*OC			−0.037^**^ (−2.41)
INS	−0.702^*^ (−1.74)	−0.785^*^ (−1.95)	−0.762^*^ (−1.9)
LOANS	−0.175 (−1.34)	−0.196 (−1.50)	−0.155 (−1.2)
PC	1.031^***^ (2.76)	1.117^***^ (2.99)	1.185^***^ (3.19)
ROA	0.417 (1.11)	0.474 (1.26)	0.586 (1.56)
ORG	1.349^***^ (9.21)	1.336^***^ (9.14)	1.322^***^ (9.08)
DEB	1.870^***^ (13.43)	1.713^***^ (12.25)	1.724^***^ (12.38)
ANALYST	0.029^***^ (10.59)	0.029^***^ (10.46)	0.028^***^ (10.18)
DUAL	−0.372^***^ (−6.85)	−0.318^***^ (−5.85)	−0.330^***^ (−6.08)
MAJOR	0.007 (0.14)	0.023 (0.47)	0.023 (0.47)
INDE	1.095^***^ (2.77)	1.405^***^ (3.55)	1.364^***^ (3.46)
IND	0.674^***^ (8.67)	0.625^***^ (8.07)	0.662^***^ (8.57)
YEAR	control		
Constant	−1.236^***^ (−5.86)	0.108^***^ (0.58)	−0.593^***^ (−2.66)
Observations	4,620	4,620	4,620
Adjusted-R^2^	0.1377	0.1430	0.1504

^∗∗∗^*p < 0.01, ^∗∗^p < 0.05, and ^∗^p < 0.1*.

The regression results also show that the coefficients of PC, ORG, DEB, ANALYST, INDE, and IND are all significantly positive. The establishment of an environment committee or a dedicated environment division can increase the firms’ responsibilities, and analyst coverage can put pressure on firms to make firms avoid risks, which can improve the quality of carbon information disclosure. INS and DUAL are negatively correlated with the quality of carbon information disclosure. LOANS, ROA, and MAJOR are not significantly correlated with the quality of carbon information disclosure in this study.

#### Robustness Tests

We carry out a number of analyses to ascertain the results’ robustness. First, to investigate whether the results are sensitive to winsorization operation, we rerun our models by winsorizing data at the 1 and 99% levels, and the results (not shown) are similar to those shown in Table 7. Second, we use an alternative measure for government regulation (PITIA). PITIA is a dummy variable that equals to 1 if the value of PITI is larger than the median and 0 otherwise. The results using this alternative measure are shown in Column 1 and Column 2 of Table 8. These results confirm Hypothesis 1 and Hypothesis 3. Third, we use business climate index as an alternative measure of executive overconfidence (OCA). OCA is a dummy variable that equals to 1 if the business climate index is greater than 100 and 0 otherwise. The value of business climate index greater than 100 indicates that executives are optimistic about the firms’ operating conditions and the macroeconomic environment (Zhang and Li, 2014). The results using this alternative measure are shown in Column 3 and Column 4 of Table 8. These results confirm Hypothesis 2 and Hypothesis 3. Fourth, we return our model by replacing the quality of carbon information disclosure with the decision of carbon information disclosure (CDIA). CDIA is a dummy variable that equals to 1 if the carbon information index is greater than 1 and 0 otherwise. This result is shown in Column 5 of Table 8. Although this result shows that there is no significant moderating effect of executive overconfidence on the relationship between government regulation and the decision of carbon information disclosure, the coefficients of government regulation and executive overconfidence are still significant at the 1% level. This result confirms Hypothesis 1 and Hypothesis 2.

**Table 8:** Robustness test results.

Variable	CDI				CDIA
	Column 1	Column 2	Column 3	Column 4	Column 5
PITIA	0.272^***^ (5.6)	0.250^***^ (5.17)			
OC		−1.486^***^ (−7.93)			−0.761^***^ (−4.92)
PITIA*OC		−0.883^**^ (−2.40)			
PITI				0.015^***^ (5.58)	0.006^***^ (3.44)
OCA			−0.967^***^ (−4.09)	−1.054^***^ (−4.40)	
PITI*OCA				−0.040^***^ (−2.74)	
PITI*OC					0.012 (0.98)
INS	−0.728 (−1.80)	−0.787^*^ (−1.96)	−0.003 (−0.55)	−0.002 (−0.43)	0.939^***^ (2.85)
LOANS	−0.166 (−1.27)	−0.139 (−1.07)	−0.242 (−1.48)	−0.223 (−1.38)	0.079 (0.74)
PC	0.993^***^ (2.65)	1.167^***^ (3.13)	1.119^**^ (2.54)	1.118^**^ (2.55)	1.064^***^ (3.51)
ROA	0.441 (1.17)	0.595 (1.59)	0.236 (0.43)	0.279 (0.52)	1.050^***^ (3.00)
ORG	1.353^***^ (9.22)	1.327^***^ (9.11)	1.003^***^ (5.85)	0.995^***^ (5.83)	1.036^***^ (7.96)
DEB	1.882^***^ (13.50)	1.729^***^ (12.38)	1.918^***^ (10.83)	1.967^***^ (11.16)	1.264^***^ (10.88)
ANALYST	0.029^***^ (10.60)	0.028^***^ (10.25)	0.027^***^ (7.92)	0.027^***^ (7.85)	0.014^***^ (6.37)
DUAL	−0.353^***^ (−6.49)	−0.312^***^ (−5.75)	−0.302^***^ (−4.59)	−0.308^***^ (−4.70)	−0.312^***^ (−6.84)
MAJOR	0.011 (0.22)	0.027 (0.54)	0.039 (0.64)	0.029 (0.47)	−0.018 (−0.45)
INDE	1.134 (2.87)	1.367 (93.47)	−0.055 (−0.11)	−0.011 (−0.02)	1.070^***^ (3.31)
IND	0.648^***^ (8.34)	0.634^***^ (8.21)	0.602^***^ (7.14)	0.628^***^ (7.49)	0.443^***^ (7.18)
YEAR	Control				
Constant	−0.657^***^ (−3.74)	−0.050 (−0.26)	0.867^***^ (2.79)	0.141 (0.41)	−1.590^***^ (−8.60)
Number of obs.	4,620	4,620	3,309	3,309	4,620
Adjusted-R^2^	0.1365	0.1487	0.1198	0.1301	

^∗∗∗^*p < 0.01, ^∗∗^p < 0.05, and ^∗^p < 0.1*.

#### Further Analysis

In China, state-owned firms have an important influence on the development of the national economy and have the mission of fulfilling their social responsibilities. According to the signaling theory, state-owned firms should actively disclose more social responsibility information. As the Chinese government pays increasing attention to environmental problems caused by climate change, a series of environmental protection policies have been issued. Compared with non-state-owned firms, state-owned firms are more likely to be directly affected by the government. Research shows that government regulation can promote the quality of carbon information disclosure of state-owned firms. For non-state-owned firms, the relationship between government regulation and carbon information disclosure is not significant (Liu and Li, 2019). In state-owned firms, executive overconfidence will increase the investment-cash flow sensitivity and increase investment. This effect of executive overconfidence is not significant in non-state-owned firms (Huang et al., 2011). Therefore, we further explore the impacts of government regulation and executive overconfidence on the quality of carbon information disclosure using samples with different ownership.

The regression results are shown in Table 9. We can find that the Government regulation (PITI) only has a significant positive impact on the quality of carbon information disclosure of state-owned firms but has no significant impact on that of non-state-owned firms. Executive overconfidence (OC) has a significant negative impact on the quality of carbon information disclosure of state-owned and non-state-owned firms, indicating that executives’ risk perception can affect the firm’s strategy and decision making, and inhibit the improvement of the quality of carbon information disclosure.

**Table 9:** Regression results of further analysis.

Variable	State-owned		Non-state-owned	
	Model 1	Model 2	Model 1	Model 2
PITI	0.032^***^ (7.53)		0.000 (0.06)	
OC		−1.811^***^ (−4.42)		−0.962^***^ (−5.27)
INS	−2.945^***^ (−3.17)	−2.855^***^ (−3.04)	0.354 (0.94)	0.332 (0.89)
LOANS	−0.230 (−0.80)	−0.332 (−1.14)	0.170 (1.35)	0.162 (1.29)
PC	3.407^***^ (4.08)	3.607^***^ (4.26)	−0.041 (−0.12)	0.032 (0.09)
ROA	−1.925 (−1.48)	−1.787 (−1.36)	0.594^*^ (1.87)	0.706^**^ (2.23)
ORG	1.428^***^ (5.56)	1.533^***^ (5.91)	0.885^***^ (5.39)	0.859^***^ (5.25)
DEB	1.645^***^ (5.16)	1.363^***^ (4.18)	1.006^***^ (7.13)	0.970^***^ (6.90)
ANALYST	0.046^***^ (7.82)	0.045^***^ (7.50)	0.023^***^ (8.48)	0.022^***^ (8.18)
DUAL	−0.448^**^ (−2.50)	−0.395^**^ (−2.18)	−0.081^*^ (−1.66)	−0.060 (−1.25)
MAJOR	0.062 (0.60)	0.056 (0.53)	−0.026 (−0.55)	−0.013 (−0.28)
INDE	1.423^*^ (1.90)	1.584^**^ (2.09)	0.249 (0.60)	0.604 (1.44)
IND	0.679^***^ (4.67)	0.589^***^ (4.01)	0.416^***^ (4.92)	0.421^***^ (5.00)
YEAR	control			
Constant	−1.849^***^ (−4.47)	0.778^*^ (1.96)	−0.278 (−1.27)	0.031 (0.16)
Number of obs.	1,598	1,598	3,022	3,022
Adjusted-R^2^	0.1430	0.1231	0.0841	0.0924

^∗∗∗^*p < 0.01, ^∗∗^p < 0.05, and ^∗^p < 0.1*.

## Conclusion, Implications, and Limitations

### Conclusion

This study examines the impacts of government regulation and executive overconfidence on the quality of firms’ carbon information disclosure. The investigation is based on data from A-share listed firms in low-carbon pilot provinces and cities in China for the 2015–2019 period. The results provide evidence that the quality of firms’ carbon information disclosure in China is generally low. Government regulation positively affects the quality of firms’ carbon information disclosure. Executive overconfidence negatively affects the quality of firms’ carbon information disclosure. Executive overconfidence negatively moderates the relationship between government regulation and the quality of firms’ carbon information disclosure. Government regulation only has significantly positive impact on the quality of carbon information disclosure of state-owned firms. Executive overconfidence has a negative impact on both state-owned and non-state-owned firms.

### Implications

On a practical note, this study has a number of implications for managers and policy makers. First, in view of the generally low quality of firms’ carbon information disclosure in China, the government should improve the environmental information disclosure polices by the development of a mandatory carbon information disclosure rule, and a guideline for firms’ carbon information disclosure to promote the quality of firms’ carbon information disclosure.

Second, the government should strengthen the regulation on firms’ environmental issue, increase penalties for firms’ environmental damage, and implement related laws and policies to promote the carbon emission reduction of firms. Meanwhile, the psychology of executive overconfidence should be paid enough attention. Executive overconfidence should be alleviated in order to mitigate the negative impact of executive overconfidence on government regulation.

Third, the firms should improve the corporate governance by establishing an environmental committee or a dedicated environment division, separating the CEO and chairman of the board, and increasing the proportion of independent directors to reduce the cost of environmental information acquisition and management to promote the quality of carbon information disclosure.

Fourth, before the mandatory carbon information disclosure policy has been implemented for all firms in China, the policy should be implemented first from state-owned firms. The state-owned firms are more sensitive to government policies, they can play a leading and exemplary role in carbon information disclosure.

### Limitations

The results of this study are subject to some limitations. The scope of our study is limited to the A-share listed firms in the low-carbon pilot provinces and cities in China. Future research can extend this study by replicating it in other economic areas, such as north America, European Union, Australia, and other developing countries. Another limitation is related to the measurement of executive overconfidence, which may not measure the psychological characteristics of executive overconfidence accurately. Additionally, with the important environmental policies issued by Chinese central government and local governments in the future, other methods can be used to explore the impact of government regulation on firms’ carbon information disclosure, such as a quasi-natural experiment.

## Data Availability Statement

The raw data supporting the conclusions of this article will be made available by the authors, without undue reservation.

## Author Contributions

RH and QY designed the studies and made a significant revision to the earlier version of the manuscript. YC and MZ assisted in research and data collection and wrote the manuscript. MZ and JL revised the manuscript. All authors contributed to the article and approved the submitted version.

## Funding

This research was funded by the Liaoning Provincial Social Science Foundation (grant no. L19BJY008).

## Conflict of Interest

The authors declare that the research was conducted in the absence of any commercial or financial relationships that could be construed as a potential conflict of interest.

## Publisher’s Note

All claims expressed in this article are solely those of the authors and do not necessarily represent those of their affiliated organizations, or those of the publisher, the editors and the reviewers. Any product that may be evaluated in this article, or claim that may be made by its manufacturer, is not guaranteed or endorsed by the publisher.
